# Genome-wide association study identifying genetic variants associated with carcass backfat thickness, lean percentage and fat percentage in a four-way crossbred pig population using SLAF-seq technology

**DOI:** 10.1186/s12864-022-08827-8

**Published:** 2022-08-15

**Authors:** Huiyu Wang, Xiaoyi Wang, Dawei Yan, Hao Sun, Qiang Chen, Mingli Li, Xinxing Dong, Yuchun Pan, Shaoxiong Lu

**Affiliations:** 1 Faculty of Animal Science and Technology, Yunnan Agricultural University, No. 95 of Jinhei Road, Kunming, 650201 Yunnan China; 2 Faculty of Animal Science, Xichang University, Xichang, 615000 Sichuan China; 3 Faculty of Agriculture and Biology, Shanghai Jiao Tong University, Shanghai, 200240 China; 4 Faculty of Animal Sciences, Zhejiang University, Hangzhou, 310058 Zhejiang China

**Keywords:** Pigs, SLAF-seq, GWAS, Carcass backfat thickness, Carcass lean percentage, Carcass fat percentage

## Abstract

**Background:**

Carcass backfat thickness (BFT), carcass lean percentage (CLP) and carcass fat percentage (CFP) are important to the commercial pig industry. Nevertheless, the genetic architecture of BFT, CLP and CFP is still elusive. Here, we performed a genome-wide association study (GWAS) based on specific-locus amplified fragment sequencing (SLAF-seq) to analyze seven fatness-related traits, including five BFTs, CLP, and CFP on 223 four-way crossbred pigs.

**Results:**

A total of 227, 921 highly consistent single nucleotide polymorphisms (SNPs) evenly distributed throughout the genome were used to perform GWAS. Using the mixed linear model (MLM), a total of 20 SNP loci significantly related to these traits were identified on ten *Sus scrofa* chromosomes (SSC), of which 10 SNPs were located in previously reported quantitative trait loci (QTL) regions. On SSC7, two SNPs (SSC7:29,503,670 and rs1112937671) for average backfat thickness (ABFT) exceeded 1% and 10% Bonferroni genome-wide significance levels, respectively. These two SNP loci were located within an intron region of the *COL21A1* gene, which was a protein-coding gene that played an important role in the porcine backfat deposition by affecting extracellular matrix (ECM) remodeling. In addition, based on the other three significant SNPs on SSC7, five candidate genes, *ZNF184*, *ZNF391*, *HMGA1*, *GRM4* and *NUDT3* were proposed to influence BFT. On SSC9, two SNPs for backfat thickness at 6–7 ribs (67RBFT) and one SNP for CLP were in the same locus region (19 kb interval). These three SNPs were located in the *PGM2L1* gene, which encoded a protein that played an indispensable role in glycogen metabolism, glycolysis and gluconeogenesis as a key enzyme. Finally, one significant SNP on SSC14 for CLP was located within the *PLBD2* gene, which participated in the lipid catabolic process.

**Conclusions:**

A total of two regions on SSC7 and SSC9 and eight potential candidate genes were found for fatness-related traits in pigs. The results of this GWAS based on SLAF-seq will greatly advance our understanding of the genetic architecture of BFT, CLP, and CFP traits. These identified SNP loci and candidate genes might serve as a biological basis for improving the important fatness-related traits of pigs.

**Supplementary Information:**

The online version contains supplementary material available at 10.1186/s12864-022-08827-8.

## Background

Carcass backfat thickness (BFT), carcass lean percentage (CLP), and carcass fat percentage (CFP) are complex quantitative traits and the most important economic factors in the pig industry. The excessive deposition of fat in pigs leads to low feed efficiency and is not favored by consumers. It is well known that CLP is strongly negatively correlated with BFT [1, 2]. In the past few years, researchers have worked on increasing carcass lean percentage and decreasing backfat thickness by advanced molecular breeding methods to improve production efficiency. To date, 3,402 QTLs and 265 QTLs associated with fatness traits and carcass lean percentage have been accumulated in the pig QTL database (http://www.animalgenome.org/cgi-bin/QTLdb/index, Dec 27, 2021).

Domestic pigs show large phenotypic variation, which is ascribed to approximately 10,000 years of natural and artificial selection [3]. At present, Chinese native pigs differ from Western commercial pigs in terms of their fatness phenotypes. Western commercial breeds, such as Large White, Landrace, and Duroc with fast growth and a high lean percentage are widely distributed all over the world. Conversely, Chinese indigenous breeds, such as the Saba pig, are fat-type pig breeds without intensive artificial selection. The native Saba pig is well known for its high prolificacy, superior meat quality, and strong resistance to harsh environments, which is widely distributed in Yunnan Province, China. However, the shared disadvantage of the indigenous breeds containing Saba pig is the excessive deposition of fat resulting in a slow growth rate and low feed conversion rate. In general, Chinese native pigs have a lean percentage of less than 45%, which is extremely different from Western commercial pigs, which typically have a lean percentage of more than 60% [4]. Using Chinese and Western pig breeds as parents, the hybrid offspring show different extreme phenotypes in fatness and more genetic variation.

Currently, remarkable advances in fatness-related traits have been made, and many related QTLs and genes have been reported [5–12]. However, identifying exact quantitative trait loci locations and new candidate genes is still a challenge. For complex traits, such as BFT, CLP and CFP, large-scale analysis is necessary to detect trait-associated SNPs. Genome-wide association study (GWAS) [13] represents a powerful approach to correlate SNPs and functional genes with quantitative traits, and has been widely applied in important economic traits of pigs, including carcass [14–19], meat quality [14, 15, 19, 20], growth [15, 21], immunity [22], and reproductive traits [23]. For species with larger genomes, such as pigs, the cost of GWAS using whole-genome sequencing (WGS) is still high at present. However, GWAS based on SNP array technology can only detect known SNP loci but not new loci. In view of these limitations, specific-locus amplified fragment sequencing (SLAF-seq) was developed, which is based on a reduced representation library and high-throughput sequencing. This technique has several distinguishing characteristics: deep sequencing, reduced sequencing costs, optimized marker efficiency, and applicability to large populations [24]. Based on the reference genome, a pre-experiment of SLAF-seq is carried out to select the appropriate combination of enzyme digestion, so as to produce a sufficient number of tags covering the whole genome and effectively avoid repeated sequences. The number of chosen fragments can be used for individualized research purposes, so as to maintain the balance between tag density and population size [24]. GWAS based on SLAF-seq was successfully used to identify SNPs associated with important economic traits in chickens, ducks, geese, and rabbits [25–30]. Additionally, genotyping and genetic structure analysis were also successfully applied in pigs using the SLAF-seq genotyping method [31, 32].

Here, we examined 223 four-way crossbreds with Landrace, Yorkshire, Duroc and Saba pigs as the hybrid parents (Saba pigs as the hybrid females) raised under the same environmental conditions for BFT, CLP and CFP traits. Subsequently, SLAF-seq was employed to perform GWAS and recover potential alleles controlling these traits. To our knowledge, this was the first SLAF-seq-based GWAS to identify SNP loci and candidate genes linked to porcine economic traits. The results provided a basis for the molecular marker-assisted breeding and improvement of the fatness-related traits in pigs.

## Results

### Phenotype description and correlation among traits

Table 1 summarized the statistical information on the seven fatness-related traits. The mean values for backfat thickness at the shoulder (SBFT), backfat thickness at the last rib (LRBFT), backfat thickness at the last lumbar (LBFT), average backfat thickness (ABFT), backfat thickness at 6–7 ribs (67RBFT), CLP, and CFP were 4.4609 cm, 2.8903 cm, 2.8371 cm, 3.399 cm, 3.6014 cm, 54.4451% and 27.1509%, respectively. All trait distributions basically conformed to the normal distribution (Fig. S1). The phenotypic correlation coefficients for the seven traits were shown in Fig. S2. Significantly positive correlations were found among five BFT traits (*r* > 0, *p* < 0.001), between five BFT traits and CFP (*r* > 0, *p* < 0.001). Five BFT traits and CFP were significantly negatively correlated with CLP (*r* < 0, *p* < 0.001). Furthermore, CLP showed the strongest negatively correlated with CFR (*r* = -0.86, *p* < 0.001), while ABFT showed the strongest positive correlations with SBFT, LRBFT and LBFT (*r* = 0.85, *p* < 0.001).

**Table 1:** Descriptive statistics of seven fatness-related traits

Traits	N^a^	Min^b^	Max^c^	Mean	SD^d^	CV^e^
Backfat thickness at the shoulder, **SBFT** (cm)	223	2.000	7.334	4.4609	0.9780	21.9442
Backfat thickness at last rib, **LRBFT** (cm)	223	1.188	5.540	2.8903	0.7842	27.1320
Backfat thickness at last lumbar, **LBFT** (cm)	223	0.974	6.232	2.8371	0.8176	28.8182
Average backfat thickness, **ABFT** (cm)	223	1.827	6.248	3.3990	0.7334	21.5764
Backfat thickness at 6–7 ribs, **67RBFT** (cm)	223	0.986	5.652	3.6014	0.8214	22.8071
Carcass lean percentage, **CLP** (%)	223	40.14	70.55	54.4451	4.4748	8.2189
Carcass fat percentage, **CFP** (%)	223	10.97	39.37	27.1509	5.1876	19.1065

^a^ Number of samples

^b^ Minimum

^c^ Maximum

^d^ Standard deviation

^e^ Coefficient of variation

### Identification of SLAFs and SNPs

**Fig. 1:**
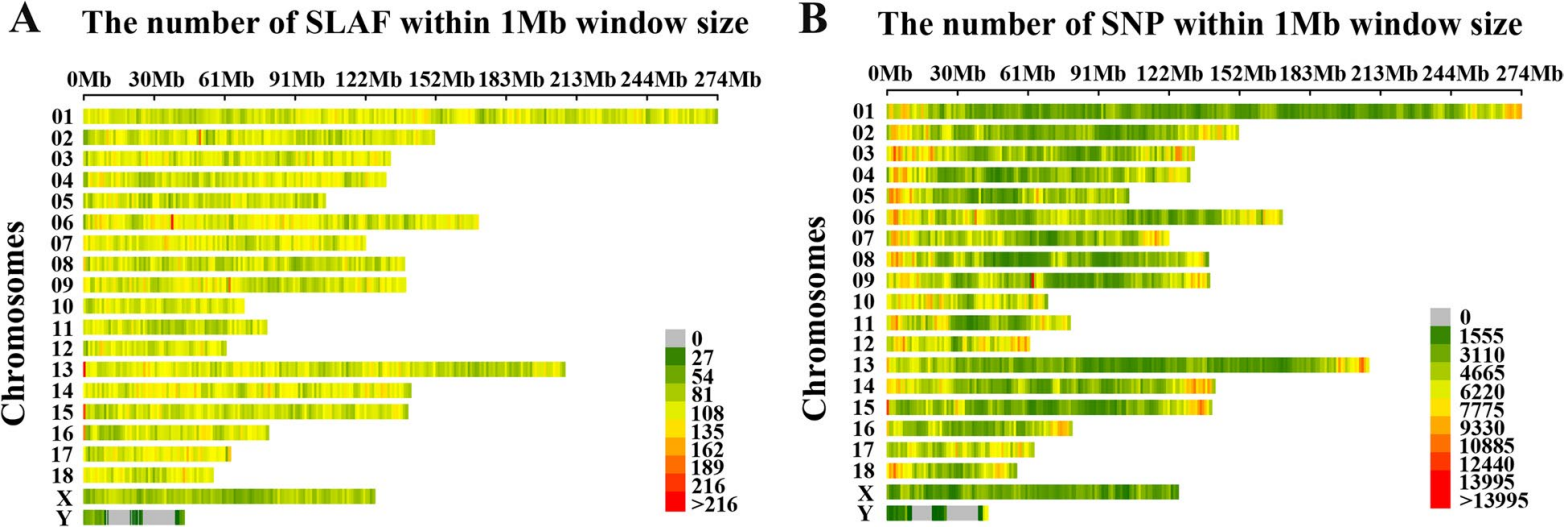
SLAF and SNP density distribution on chromosomes of the pig genome. **A** The number of SLAFs within 1 Mb window size. **B** The number of SNPs within 1 Mb window size. The horizontal axis (X-axis) shows the chromosome length (Mb). Color index indicates the number of labels

According to the selection principle of the enzyme digestion scheme, two restriction enzymes, RsaI and HaeIII, were selected as enzyme combinations for developing SLAF tags, and the sequence with the length of 314–344 bp was defined as SLAF tags. A total of 1,109.92 million reads were obtained from all individuals. Average Q30 and GC contents were 90.74% and 44.83%, respectively. Similar to the number of expected SLAFs, a total of 1,552,377 SLAF tags (an average of 331,608 SLAFs for each individual) were identified from all individuals with sequencing to an 11.94 average depth. Furthermore, 245,734 SLAFs were identified across the whole genome through genomic mapping, of which 230,239 polymorphic SLAF tags (Table S1). In addition, *Oryza sativa indica* was used as a control during sequencing. The results showed that the percentage of digestion normally and paired-end mapped reads of control were 90.77% and 95.4%, respectively, indicating that the SLAF-seq process was normal. The density distribution of SLAFs was calculated throughout the pig genome and was shown in Fig. 1A.

After genomic mapping and SNP calling, a total of 10,784,484 SNPs were discovered using all individuals. A series of quality control filtering of SNPs was performed to identify 227,921 highly consistent SNPs used in the further analysis based on the selection criteria (integrity > 0.8; MAF > 0.05). The density distribution of SNPs was calculated throughout the pig genome and was shown in Fig. 1B. Almost all of the genome’s non-overlapped 1 Mb regions contained SNPs, which indicated that the data was reliable.

### Genome-wide association study and identification of candidate genes

**Fig. 2:**
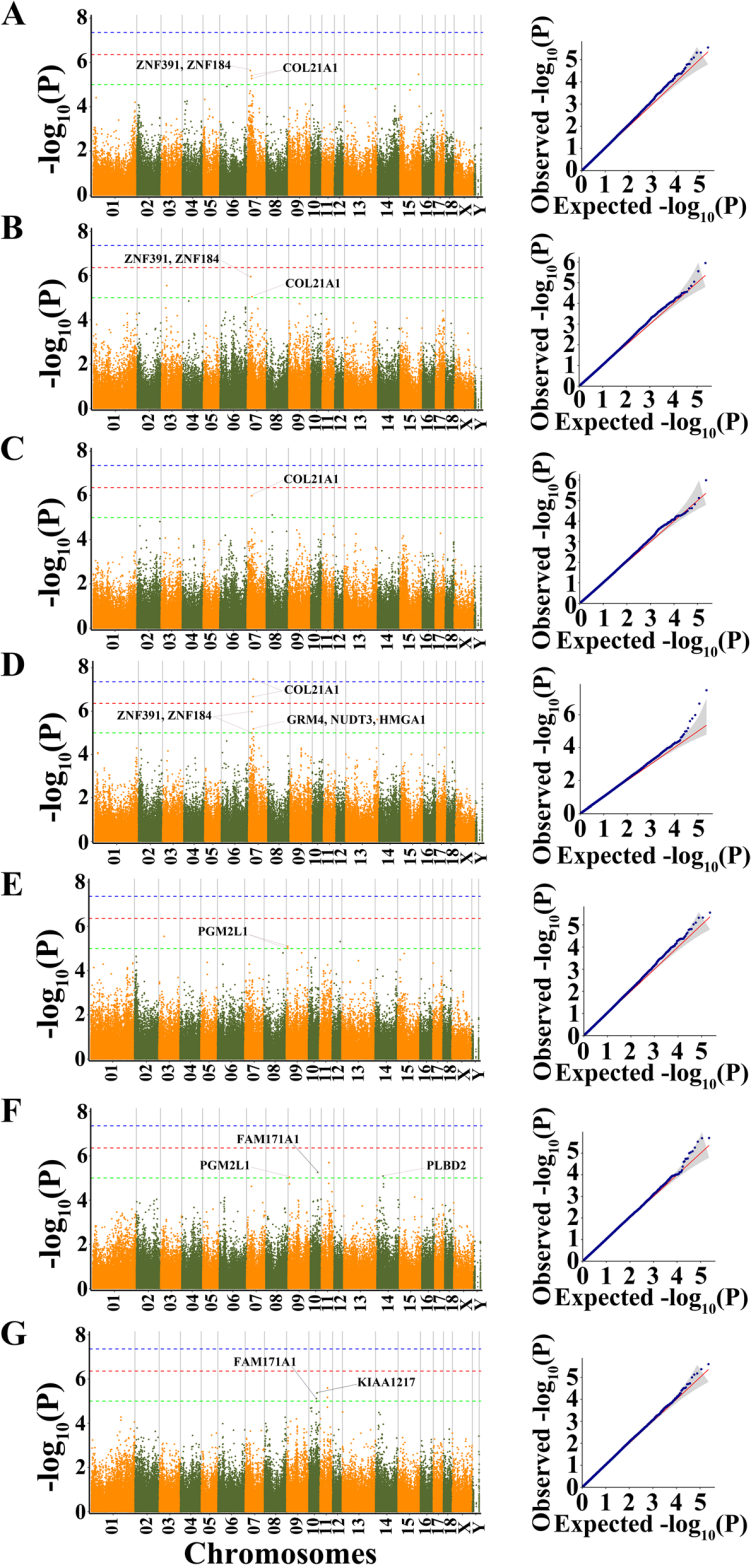
Manhattan plots and QQ plots for seven fatness-related traits using MLM model of GEMMA software. **A** SBFT **B** LRBFT **C** LBFT **D** ABFT **E** 67RBFT **F** CLP **G** CFP. Negative log_10_ (*P*) values of the filtered high-quality SNPs were plotted against their genomic positions. The dashed lines of green, orange and blue correspond to the Bonferroni-corrected thresholds of *P* = 1.00 × 10^–5^ (-log_10_(*P*) = 5), *P* = 4.39 × 10^–7^(-log_10_(*P*) = 6.36) and *P* = 4.39 × 10^–8^ (-log_10_(*P*) = 7.36), respectively

As population stratification might affect GWAS, quantile–quantile (Q-Q) plots of all traits were drawn. The observed *P*-value calculated by the association study fit the expected ones, which suggested that the population stratification was well-corrected and the association analysis using MLM was reliable. The Q-Q plot of each trait was shown following the Manhattan plot of the corresponding trait (Fig. 2). A total of 20 SNPs were identified as significant (P ≤ 1.0 × 10^–5^) for the traits investigated (Table 2). The phenotypic variation explained (PVE) by the significant SNPs was from 4.588 to 16.855. Among the significant SNPs, only one SNP on SSC7 (SSC7:29,503,670 for ABFT) exceeded the 1% genome-wide significance level (*P* = 3.26 × 10^–8^). Another SNP in proximity to this SNP (rs320451735 for ABFT) exceeded the 10% genome-wide significance level (*P* = 2.21 × 10^–7^). Among the detected SNPs, four, three, two, seven, five, six and four SNPs, were significantly associated with SBFT, LRBFT, LBFT, ABFT, 67RBFT, CLP and CFP traits, respectively. These SNPs detected were distributed in ten *Sus scrofa* chromosomes (SSC), including SSC3, SSC7, SSC8, SSC9, SSC10, SSC11, SSC12, SSC13, SSC14 and SSC15. In this study, 33 genes located within 100 kb upstream and downstream of these significant SNPs were considered potential candidate genes (Table 2).

**Table 2:** The significant SNPs and candidate genes for seven fatness-related traits

Trait^a^	SNP^b^	Pos (bp)^c^	MAF^d^	*P*-value^e^	-log_10_P^f^	Allele	PEV (%)^g^	Genes^h^	Distance^i^
**SBFT**		SSC7:21,392,136	0.11	2.31 × 10^–6^	5.64	T/C	11.182	*POM121L2*	U:50,008
								*ZNF391*	Intron
								*ZNF184*	D:21,214
	rs1112937671	SSC7:29,486,003	0.12	5.29 × 10^–6^	5.28	T/C	10.845	*COL21A1*	Intron
		SSC7:29,503,670	0.14	3.94 × 10^–6^	5.4	T/C	10.549	*COL21A1*	Intron
	rs319334375	SSC15:117,022,248	0.2	3.42 × 10^–6^	5.47	T/C	8.982	*BARD1*	D:25,591
								*ENSSSCG00000048197*	U:30,544
**LRBFT**	rs320451735	SSC7:21,466,553	0.12	1.11 × 10^–6^	5.96	C/A	15.435	*ZNF391*	U:66,090
								*ZNF184*	U:31,328
		SSC7:29,503,670	0.14	8.98 × 10^–6^	5.05	T/C	12.749	*COL21A1*	Intron
	rs332294996	SSC3:34,308,396	0.06	2.80 × 10^–6^	5.55	G/A	8.402	NA	NA
**LBFT**		SSC7:29,503,670	0.14	1.02 × 10^–6^	5.99	T/C	14.006	*COL21A1*	Intron
	rs322063364	SSC8:36,710,706	0.08	7.50 × 10^–6^	5.12	G/T	11.22	NA	NA
**ABFT**		AEMK02000449.1:179,574	0.12	1.73 × 10^–6^	5.76	C/G	6.138	*OR4P4L*	U:56,372
								*OR4C13L*	U:69,382
								*OR5D14*	D:96,469
								*OR5D13L*	D:83,741
		SSC7:21,392,136	0.11	1.01 × 10^–5^	5.00	T/C	12.82	*POM121L2*	U:50,008
								*ZNF391*	Intron
								*ZNF184*	D:21,214
	rs320451735	SSC7:21,466,553	0.12	1.05 × 10^–6^	5.98	C/A	14.59	*ZNF391*	U:66,090
								*ZNF184*	U:31,328
	rs1112937671	SSC7:29,486,003	0.12	2.12 × 10^–7^*	6.67*	T/C	15.416	*COL21A1*	Intron
		SSC7:29,503,670	0.14	3.26 × 10^–8^ **	7.49**	T/C	16.855	*COL21A1*	Intron
	rs341689410	SSC7:30,292,654	0.14	6.58 × 10^–6^	5.18	G/A	12.635	*GRM4*	U:13,533
								*SMIM29*	D:36,896
								*NUDT3*	D:27,653
								*HMGA1*	D:27,800
	rs706606912	SSC13:197,751,407	0.08	2.46 × 10^–6^	5.61	G/A	8.815	*MRPS6*	Intron
								*SLC5A3*	U:32,711
**67RBFT**		AEMK02000449.1:179,574	0.12	4.84 × 10^–6^	5.32	C/G	7.11	*OR4P4L*	U:56,372
								*OR4C13L*	U:69,382
								*OR5D14*	D:96,469
								*OR5D13L*	D:83,741
	rs341161412	SSC9:8,763,434	0.38	8.00 × 10^–6^	5.1	C/G	10.151	*LIPT2*	D:74,668
								*KCNE3*	D:47,288
								*PGM2L1*	UPS
								*P4HA3*	U:95,660
								*ENSSSCG00000042110*	U:60,242
	rs343149423	SSC9:8,763,503	0.38	9.62 × 10^–6^	5.02	A/G	10.01	*LIPT2*	D:74,599
								*KCNE3*	D:47,219
								*PGM2L1*	UPS
								*P4HA3*	U:95,729
								*ENSSSCG00000042110*	U:60,311
	rs332294996	SSC3:34,308,396	0.06	2.81 × 10^–6^	5.55	G/A	5.416	NA	NA
	rs344553616	SSC12:50,896,936	0.11	4.80 × 10^–6^	5.32	G/A	9.537	*PITPNM3*	3’UTR
								*PIMREG*	DOWNS
								*AIPL1*	D:6,502
**CLP**		AEMK02000598.1:813,208	0.38	2.95 × 10^–6^	5.53	T/C	14.179	*OR1J4L*	DOWNS
								*OR1J4L*	D:88,150
		SSC9:8,744,721	0.08	9.18 × 10^–6^	5.04	C/T	13.264	*LIPT2*	D:93,381
								*KCNE3*	D:66,001
								*PGM2L1*	Intron
								*P4HA3*	U:76,947
								*ENSSSCG00000042110*	U:41,529
		SSC14:38,533,013	0.25	8.13 × 10^–6^	5.09	G/A	4.588	*SDS*	U:19,133
								*LHX5*	U:75,836
								*SDSL*	U:48,858
								*PLBD2*	Intron
								*DTX1*	D:27,847
								*RASAL1*	D:65,256
	rs320036825	SSC10:46,524,739	0.45	5.52 × 10^–6^	5.26	G/A	8.98	*FAM171A1*	Intron
	rs329489266	SSC11:47,266,057	0.5	2.01 × 10^–6^	5.7	C/G	11.239	NA	NA
	rs81261044	SSC11:47,266,119	0.42	1.98 × 10^–6^	5.7	G/A	10.719	NA	NA
**CFP**	rs320036825	SSC10:46,524,739	0.45	7.61 × 10^–6^	5.12	G/A	10.873	*FAM171A1*	Intron
	rs329489266	SSC11:47,266,057	0.5	6.72 × 10^–6^	5.17	C/G	9.643	NA	NA
	rs81261044	SSC11:47,266,119	0.42	2.51 × 10^–6^	5.6	G/A	10.736	NA	NA
		SSC10:50,888,840	0.4	4.26 × 10^–6^	5.37	T/G	9.072	*KIAA1217*	Intron

^a^ Description of the traits is in Table 1*SBFT* Backfat thickness at the shoulder *LRBFT* Backfat thickness at the last rib *LBFT* Backfat thickness at the last lumbar *ABFT* Average backfat thickness *67RBFT* Backfat thickness at 6–7 ribs *CLP* Carcass lean percentage *CFP* Carcass fat percentage

^b^ SNP rs ID from Ensembl

^c^ Positions of the significant SNP according to the *Sus Scrofa* Build 11.1 assembly *SSC Sus Scrofa* chromosome

^d^ Minor Allele Frequency

^e^ Genome-wide significant associations are underlined ^e,f^*and** represented the 10% and 1% genome-wide significance, respectively

^g^ Phenotypic Variation Explain

^h^ The gene located with 100 kb upstream and downstream of the significant SNP

^i^ *UPS* Upstream (5’ of the gene) *DOWNS* Downstream (3’ of the gene) *U*/*D* represented the gene located upstream or downstream of the SNP (Intergenic region)

Interestingly, nine significant SNPs were identified to be associated with at least two fatness-related traits (Table S2). Among them, four SNPs on SSC7 were identified to be associated with at least two BFTs (Table S2). Two SNPs (SSC7:21,392,136 and rs320451735) on SSC7 have located apart 74.417 kb each other, while another two (rs1112937671 and SSC7:29,503,670) on SSC7 have located apart 17.667 kb each other, which meant that the responsible gene may locate near here. The results showed that the nearest genes of the SSC7:21,392,136 and rs320451735 were *ZNF184* and *ZNF391*, while rs1112937671 and SSC7:29,503,670 were located within an intron region of the *COL21A1* gene (Table S2, Table 2). Furthermore, a significant SNP (rs341689410) on SSC7 had located downstream 13.533 kb, upstream 27.653 kb and 27.8 kb of *GRM4, NUDT3* and *HMGA1*, respectively. On SSC9, two adjacent SNPs (rs341161412 and rs343149423) associated with 67RBFT and another adjacent SNP (SSC9:8,744,721) related to CLP were located in a region between 8.744 Mb and 8.763 Mb (19 kb interval). These three SNPs were located in the *PGM2L1* gene. (Table 2). In addition, it was interesting that two significant SNPs on SSC11 and one significant SNP on SSC10 were found to have pleiotropic effects on CLP and CFP. The locus on SSC10 was located in the intron region of the *FAM171A1* gene. Nevertheless, there were no genes found nearby the two loci on SSC11 (Table S2, Table 2). Finally, two significant SNPs (SSC14:38,533,013 for CLP and SSC10:50,888,840 for CFP) were located in the intron region of *PLBD2* and *KIAA1217*, respectively (Table 2).

### Comparison with previously mapped QTL in pigs

To evaluate whether QTLs associated with seven fatness-related traits in this study replicate any previously known QTLs, the Pig Quantitative Trait Locus (QTL) Database (Pig QTLdb, https://www.animalgenome.org/cgi-bin/QTLdb/SS/index) was searched based on SNP and QTL locations. A total of 20 SNPs were identified in the study, of which 10 SNPs were located in previously reported QTL regions in pigs. The remaining 10 SNPs had not been included in any previously reported QTLs that were associated with BFT, CLP and CFP of pigs. On SSC7, a total of five SNPs significantly associated with BFT were found, which were located in a region from 21.39 to 30.29 Mb (8.9 Mb interval). This region was located within 20 previously reported QTLs associated with BFT. Among five significant SNPs, three adjacent SNPs (from 29.49 to 30.29 Mb, 0.8 Mb interval) were located within the 26 BFT-related QTLs that had been previously reported. On SSC9, a total of three adjacent SNPs, including two SNPs associated with 67RBFT and one SNP associated with CLP were located in a region between 8,745 and 8,764 kb (19 kb interval), which was located in one formerly acknowledged QTL (from 0.1 to 11.1 Mb) associated with LRBFT and CLP. Besides, one SNP locus (rs344553616) on SSC12 associated with 67RBFT was located within a known QTL region (47.9–59.4 Mb) related to backfat thickness at 10 ribs (10RBFT), another locus (rs319334375) on SSC15 associated with SBFT was located within a formerly reported QTL (57.5–120.1 Mb) related to SBFT. These results were shown in Table S3.

### GO annotation of candidate genes

The result of GO annotation showed that *ZNF184* and *ZNF391* were mainly involved in regulation of transcription, DNA-template and nucleic acid binding. The cellular components of *COL21A1* were collagen trimer and extracellular matrix. *PGM2L1* mainly participated in carbohydrate metabolic process, phosphorylation and glucose-1,6-bisphosphate synthase activity. The *PLBD2* gene was involved in lipid catabolic process and hydrolase activity. GO annotation results of other genes were shown in Table S4.

## Discussion

### Comparison of SLAF-seq with other genotyping methods

In the present study, we performed a GWAS based on SLAF-seq to screen and select candidate SNPs for fatness-related traits on 223 four-way crossbreds with Landrace, Yorkshire, Duroc and Saba pigs as the hybrid parents. A total of 1,552,377 SLAFs were predicted, and 10,784,484 SNPs were obtained (11.94-fold sequencing depth), and 227, 921 highly consistent SNPs were evenly distributed over the entire genome (As shown in Fig. 1B, almost all of the genome’s non-overlapped 1 Mb regions contained SNPs) were identified to perform GWAS, which were nearly three times the number of SNPs on Illumina PorcineSNP80 Genotyping BeadChip. SLAF-seq has a more significant advantage for genome-wide association studies compared with SNP arrays and can produce more information on genomic variation and allow the detection of novel SNPs on the genome. Currently, SLAF-seq was successfully applied to pig genotyping and identified a large number of new mutation sites [31, 32]. Besides, SLAF-seq can be applied not only to species with a reference genome but also to those without a reference genome. So far, SLAF-seq was successfully used to create a genetic map for common carp (Cyprinus carpio L.), soybean and orchardgrass [24, 33, 34]. However, compared with WGS, which is another major genotyping method that has been used over the last several years, SLAF-seq as a reduced representation sequencing method hasn’t covered the whole genome SNPs. For large populations, WGS is prohibitively expensive, while SLAF-seq can reduce sequencing costs. In the study, the pre-experiment of SLAF-seq was based on the pig reference genome to screen the appropriate enzyme digestion combination (RsaI and HaeIII), which ensured the number and depth of SLAF tags covering the whole genome (Table S1, Fig. 1A), and effectively avoided repeated sequences. Considering the huge differences in genomic variation between Chinese and Western pig breeds, SLAF-seq used in the GWAS study for large populations was a better choice in the current higher sequencing cost and had great potential for further study in more pig breeds. Therefore, the SLAF-seq method could be considered a more competitive choice in pig genome research at present.

### Comparison of QTLs identified in this study with findings of previous studies

In the study, a genome-wide association study was performed for seven fatness-related traits to identify significant SNPs in pigs. Of the 20 significant SNPs associated with BFT, CLP and CFP traits found in four-way crossbred pigs in our study, except for ten significant SNPs that had not been reported in previous studies, other SNPs were located in previously reported QTLs. On SSC7, a total of five SNPs significantly associated with BFTs were located in a region (from 21.39 to 30.29 Mb, 8.9 Mb interval), which was located within 20 previously reported QTLs associated with BFT (Table S3), which span more than 16.4 Mb. We further narrowed the interval of QTLs for BFT in the study. Among five significant SNPs, three adjacent SNPs (from 29.49 to 30.29 Mb, 0.8 Mb interval) were located in the 26 BFT-related QTLs that had been previously reported. Qiao et al. found that the strongest association was between a 750 kb region (between 34. 67 and 35. 42 Mb for Sscrofa10.2) on SSC7 and backfat thickness at the first rib [7]. Gozalo-Marcilla et al. found significant genome-wide associations with backfat thickness for 13 SNPs in genomic regions on SSC7 at 30 Mb (30.10–30.89 Mb) in three lines [35]. Thus, the 8.9 Mb region (between 21.39 and 30.29 Mb) on SSC7, especially the 0.8 Mb region (between 29.49 and 30.29 Mb) might be important QTLs related to BFT. On SSC9, there were three adjacent SNPs, including two SNPs associated with 67RBFT and one SNP associated with CLP, located in the region of 8,745 to 8,764 kb (19 kb interval), which was located in one previously reported QTL (from 0.1 to 11.1 Mb) associated with LRBFT and CLP. Gozalo-Marcilla et al. also found significant genome-wide associations with backfat thickness for 51 SNPs in genomic regions on SSC11 at 8 Mb (7.03–9.57 Mb) in four lines [35].

### Candidate genes for fatness-related traits

The GWAS result showed that four significant SNPs on SSC7 were associated with at least two BFTs. The nearest genes of two SNPs (SSC7:21,392,136 and rs320451735) were zinc finger protein 184 (*ZNF184*) and zinc finger protein 391 (*ZNF391*). The result of GO annotation showed that *ZNF184* and *ZNF391* participated in regulation of transcription, DNA-template and nucleic acid binding. As is known, zinc-finger proteins (ZFPs) represent the largest transcription factor family in mammals [36]. These two genes are members of the ZFP transcription factor family, which are essential for controlling a variety of growth and development processes through nucleic acid binding and transcription activation [37]. An increasing number of ZFPs involved in adipogenesis had been discovered, such as zinc finger protein 423 (Zfp423) [38, 39], Zfp467 [40, 41], Zfp521 [42], ZNF395 [43]. Besides, a large number of ZFPs played roles in preadipocyte differentiation, such as ZNF638 [44], GATA2 [45], GATA3 [45] and SLUG [46], which belong to the subfamily of C2C2-type zinc finger proteins, which are characterized by a highly conserved zinc finger DNA binding domain. It is known that *ZNF184* and *ZNF391* also are the C2H2-type zinc finger transcriptional factors. According to their structure and function, it was inferred that *ZNF184* and *ZNF391* might be involved in regulating adipogenesis as C2H2-type transcriptional factors. In addition, another two SNPs (rs1112937671 and SSC7:29,503,670) on SSC7 were located within an intron region of collagen type XXI alpha 1 chain (*COL21A1*). The result of GO annotation showed that the cellular component of *COL21A1* was collagen trimer and extracellular matrix (ECM). It has been reported that *COL21A1* is VAdomain-containing collagen with a domain structure and which is a part and the smallest of the FACIT family of collagen expressed in various tissues [47, 48]. By connecting them to other matrix components or cells, the co-expression of collagen XXI and collagen I in tissues and muscles plays a significant role in the organization of interstitial collagen fibrils [47, 49]. The collagen protein has an important role in ECM remodeling [50], which is associated with the modulation of adipogenesis during adipose tissue expansion [51]. Several research showed that collagen I genes (*COL1A1* and *COL1A2*) influenced porcine fat deposition by affecting ECM remodeling [52, 53]. Thus, it was inferred that *COL21A1* might play an important role in porcine backfat deposition by affecting ECM remodeling and should be considered a strong candidate gene for porcine BFT traits.

Furthermore, a significant SNP (rs341689410) on SSC7 had located upstream 13.5 kb, downstream 27.6 kb and 27.8 kb of glutamate metabotropic receptor 4 (*GRM4*), nudix hydrolase 3 (*NUDT3*) and high mobility group protein HMG-I (*HMGA1*), respectively. GO annotation result showed that *GRM4* was involved in activation of MAPK activity (Table S4). The MAPK pathway was demonstrated in numerous studies to be crucial for adipogenesis [54, 55]. So *GRM4* might play an important role in adipogenesis by activation of MAPK activity. A study found that variants of *NUDT3* have been linked to alterations in human body mass index values [56]. As above, the two genes might be potential candidate genes for the SSC7 locus. Besides, given that *HMGA1* is functionally related to fat metabolism and that several of its variations have been linked to the backfat thickness [7], it may be a leading candidate gene for the locus. Some research suggested that *HMGA1* could act as an *IGF1* activity regulator to control the uptake of glucose [57] and could bind to PPARG, a crucial regulator of adipocyte differentiation and glucose homeostasis [58]. On SSC9, two adjacent SNPs (rs341161412 and rs343149423) associated with 67RBFT and another adjacent SNP (SSC9:8,744,721) related to CLP were located in a region between 8.744 and 8.763 Mb (19 kb). These three SNPs were located in the phosphoglucomutase 2 like 1 (*PGM2L1*) gene. GO annotation result showed that *PGM2L1* was mainly involved in carbohydrate metabolic process, phosphorylation and glucose-1,6-bisphosphate synthase activity. It is known that *PGM2L1*, a member of a distinct family of α-phosphohexomutases widely distributed in prokaryotes, is able to produce glucose-1,6-bisphosphate, a crucial metabolic regulator. According to a study, *PGM2L1* encodes a crucial enzyme that is essential for glycogen metabolism, glycolysis, and gluconeogenesis [59]. Therefore, *PGM2L1* should be considered a potential candidate gene for BFT and CLP traits.

For the traits of CLP and CFP, one significant SNP locus (rs320036825) onSSC10 was located in an intron region of family with sequence similarity 171 member A1 (*FAM171A1*). The result of GO annotation showed that the *FAM171A1* gene participated in regulation of cell shape (Table S4). The *FAM171A1* gene is a member of the family of sequence similarities, which is responsible for encoding the APCN/FAM171A1 protein. APCN/FAM171A1 is an evolutionarily conserved 98 kDa transmembrane type I glycoprotein, which is expressed in various cells and participates in the regulation of cytoskeleton dynamics, thereby regulating cell shape [60]. A study found that the related gene *FAM110B* was associated with the lean meat percentage of pigs [12]. According to its function and extensive expression in various cells, perhaps *FAM171A1* could be considered a potential candidate gene for the porcine CLP and CFP traits. Additionally, one significant SNP on SSC14 associated with CLP was located within the intron region of phospholipase B-like 2 (*PLBD2*). GO annotation result indicated that the *PLBD2* gene participated in lipid catabolic process. Wang et al. found that *PLBD2* was predominantly involved in the lipid catabolic process, which was consistent with the favoured selection for fatness in Chinese pigs [61]. Thus, the *PLBD2* gene should be considered a strong candidate gene for porcine CLP trait. Finally, one SNP (SSC10:50,888,840) related to CFP trait was located in an intron region of *KIAA1217*. A study found that the *KIAA1217* gene showed differences in promoter methylation and mRNA expression in the omental visceral adipose tissue between non-obese and obese individuals [62]. Perhaps, the *KIAA1217* gene might also be used as a potential candidate gene for the porcine CFP trait.

However, these identified loci and genes need to be further verified in more pig populations, and their functions also need to be validated by more biological experiments in pigs.

## Conclusions

We performed GWAS based on SLAF-seq for seven fatness-related traits in 223 four-way crossbred pigs. The sequencing results showed that the SLAF-seq method could be considered a more competitive choice in pig genome research at present. Two regions on SSC7 (from 21.39 to 30.29 Mb, 8.9 Mb) and SSC9 (from 8,745 to 8,764 kb, 19 kb) were found to be associated with fatness-related traits. Furthermore, a total of eight candidate genes, including *COL21A1*, *ZNF184*, *ZNF391*, *HMGA1*, *GRM4*, *NUDT3*, *PGM2L1*, and *PLBD*2 were proposed for fatness-related traits. The GWAS using SLAF-seq data provided new insight into the genetic characteristics of fatness-related traits in pigs. Moreover, these identified SNP loci and candidate genes might serve as a biological basis for improving these important fatness-related traits of pigs.

## Materials and methods

### Experimental animals and phenotypes

A total of 223 four-way crossbred pigs (108 males and 115 females, DSYLS) used in this study were produced with 7 hybrid boars (Duroc × Saba, DS) and 37 hybrid sows (Yorkshire × (Landrace × Saba), YLS) as the parents. All the animals were reared under the same nutritional and environmental conditions until slaughtered (105.25 ± 15.75 kg) at the pigs and broilers breeding farm in Chuxiong City, Yunnan Province, China. Ear tissue samples were collected from 223 crossbreds.

In the study, the studied phenotypes were BFT, CLP and CFP traits. BFT traits included backfat thickness at the shoulder (SBFT), backfat thickness at the last rib (LRBFT), backfat thickness at the last lumbar (LBFT), average backfat thickness (ABFT), and backfat thickness at 6–7 ribs (67RBFT). The SBFT was the backfat thickness at the thickest point over the shoulder. The LRBFT was the backfat thickness at the last rib. The LBFT was the backfat thickness at the last lumbar. The 67RBFT was the backfat thickness between the 6th and 7th ribs. SBFT, LRBFT, LBFT and 67RBFT were measured using the vernier caliper. The ABFT was calculated as the average of three measurements: SBFT, LRBFT, and LBFT. The left side of each carcass was dissected by separating bone, muscle, fat, and skin. Each component was individually weighed and recorded as bone weight (BW), muscle weight (MW), fat weight (FW) and skin weight (SW). CLP and CFP were calculated as follows:

$$\mathrm{CLP }(\mathrm{\%}) =\mathrm{MW}/(\mathrm{BW}+\mathrm{MW}+\mathrm{FW}+\mathrm{SW})$$

$$\mathrm{CFP }(\mathrm{\%}) =\mathrm{FW}/(\mathrm{BW}+\mathrm{MW}+\mathrm{FW}+\mathrm{SW})$$

The MEANS procedure of SAS (SAS Institute, Inc., Cary, NC) was used to generate descriptive statistics for studied traits. The sample distribution was visualized as a frequency distribution histogram using the R package “ggpubr”. The phenotypic correlation was visualized as a correlation heatmap by the R function “PerformanceAnalytics”.

### SLAF library construction and sequencing

Genomic DNA was extracted from ear tissue samples. Total DNA was extracted by the phenol–chloroform extraction method, with concentration and purity measured using the NanodropTM 2000 spectrophotometer (Thermo Scientific, Waltham, MA, USA) and electrophoresis. A simulated restriction enzyme digestion was carried out on the current pig genome (Sscrofa 11.1, ftp://ftp.ensembl.org/pub/release-102/) to identify expected SLAF yield, avoid repetitive SLAFs, and obtain the relatively uniform distribution of restriction fragments in the genome. As a result, genomic DNA was digested with RsaI and HaeIII restriction enzyme combinations. Meanwhile, to assess the experimental procedure, Oryza sativa indica (http://rapdb.dna.affrc.go.jp/) was used as a control for evaluating the effectiveness of enzyme digestion and paired-end mapped reads. In brief, SLAF library construction and sequencing for each individual was conducted as described previously [24] with slight modifications: DNA fragments of 314–344 base pair (bp) were selected as SLAFs and used for paired-end sequencing by Illumina HiSeq 2500 system (Illumina, Inc., San Diego, CA, USA) at Beijing Biomarker Technologies Corporation. Raw sequencing reads were identified by dual-indexing [63] and classified to each sample.

### Genome mapping, SNP calling and filtering

Raw paired-end reads were mapped to the pig reference genome (Sscrofa 11.1_102) using BWA software [64]. Local realignments were conducted, and SNPs were detected using GATK software [65]. To ensure the accuracy of the SNPs identified using GATK, SAMtools software also was used to detect SNPs [66]. The intersection of SNPs detected using the two methods was designated as the final SNPs. Ultimately, highly consistent SNPs were obtained for GWAS analysis by filtering according to minor allele frequency (MAF: 0.05) and integrity (int: 0.8) using PLINK 2 [67].

### Genome-wide association study (GWAS)

Total filtered SNPs (integrity > 0.8, MAF > 0.05) detected from 223 accessions were used for GWAS. GEMMA software [68] was used for association analysis between traits and SNPs. The mixed linear model (MLM) formula of GEMMA software was as follows:

$$\mathrm{y }=\mathrm{ W\alpha }+\mathrm{ X\beta }+\mathrm{ Z\mu }+\upvarepsilon$$

where y was the phenotype, X was the genotype, W (fixed effect) was the matrix of population structure calculated by the ADMIXTURE software [69], and Z was the matrix of kinship relationship calculated using GCTA software [70], Wα and Xβ were fixed effects, Zμ and ε were random effects. Finally, an association result could be obtained for each variation site. Since the Bonferroni correction (BC) method [68] for multiple testing was too conservative and only a few significant SNPs were detected, markers with adjusted − log10 (P) ≥ 5 (control threshold) were regarded to be significant SNPs for fatness-related traits. Based on the number of SNPs analyzed (*n* = 227,921), the threshold *p*-value for genome-wide 1% and 10% significance were 4.39 × 10^–8^ (0.01/227,921) and 4.39 × 10^–7^ (0.1/227,921), respectively. Considering the complexity of the traits, markers that passed the threshold score or above the threshold − log_10_(P) were held to be significantly associated with the target trait.

### Identification and functional enrichment analysis of candidate genes

Based on the reference [71–73], the genes within 100 kb up- or down-stream of significant associated SNPs were considered trait-associated potential candidate genes. The relevant information of genes in 100 kb windows surrounding each significant SNP was downloaded from the Ensembl Sscrofa11.1 database (www.ensembl.org). GO annotation of candidate genes was then performed using Gene Ontology Consortium (http://geneontology.org).

## Supplementary Information

**Additional file 1: Figure S1.** Frequency distribution histogram for seven fatness-related traits, including five BFTs, CLP and CFP. **A** SBFT. **B** LRBFT. **C** LBFT. **D** ABFT. **E** 67RBFT. **F** CLP. **G** CFP.

**Additional file 2: Figure S2.** The phenotypic correlation for seven fatness-related traits, including five BFTs, CLP and CFP. The values in the box represented the phenotypic correlation of the traits. Negative values represented negative correlation, and positive values represented positive correlation. *significant at *P*<0.05, **significant at *P*<0.01, ***significant at *P*<0.001. All of the phenotypic correlation coefficients were significant with *P* < 0.05.

**Additional file 3: Table S1.** Distribution of SLAF tags and polymorphism SLAF tags on Sus Scrofa chromosomes.

**Additional file 4: Table S2.** The information of shared SNPs for fatness-related traits.

**Additional file 5: Table S3.** Comparison of significant SNPs with previously reported QTLs from the pig QTL database and newly significant SNPs for fatness-related traits.

**Additional file 6: Table S4.** The description and GO annotation of the gene with 100 kb upstream and downstream of the significant SNP.

## Data Availability

The Genome sequencing raw data was deposited in NCBI’s SRA database (https://trace.ncbi.nlm.nih.gov/Traces/sra/sra.cgi? view = studies&f = study&term = &go = Go; Accession: SRP376933).

## Abbreviations

GWAS: Genome-wide association study
SLAF-seq: Specific-locus amplified fragment sequencing
SNP: Single nucleotide polymorphism
QTL: Quantitative trait loci;
BFT: Carcass backfat thickness
SBFT: Backfat thickness at the shoulder
LRBFT: Backfat thickness at the last rib
LBFT: Backfat thickness at the last lumbar
ABFT: Average backfat thickness
67RBFT: Backfat thickness at 6–7 ribs
CLP: Carcass lean percentage
CFP: Carcass fat percentage
SSC: Sus scrofa Chromosome
MAF: Minor Allele Frequency
Q-Q plot: Quantile–quantile plot
MLM: Mixed linear model
PVE: Phenotypic variation explain
GO: Gene ontology
WGS: Whole-genome sequencing
ZFP: Zinc-finger protein
ECM: Extracellular matrix
