# Supplementary figures and images for: Genome-wide association study identifying genetic variants associated with carcass backfat thickness, lean percentage and fat percentage in a four-way crossbred pig population using SLAF-seq technology
Source: BMC Genomics. 2022 Aug 15;23:594. doi: 10.1186/s12864-022-08827-8 (PMC9380336; doi:10.1186/s12864-022-08827-8)

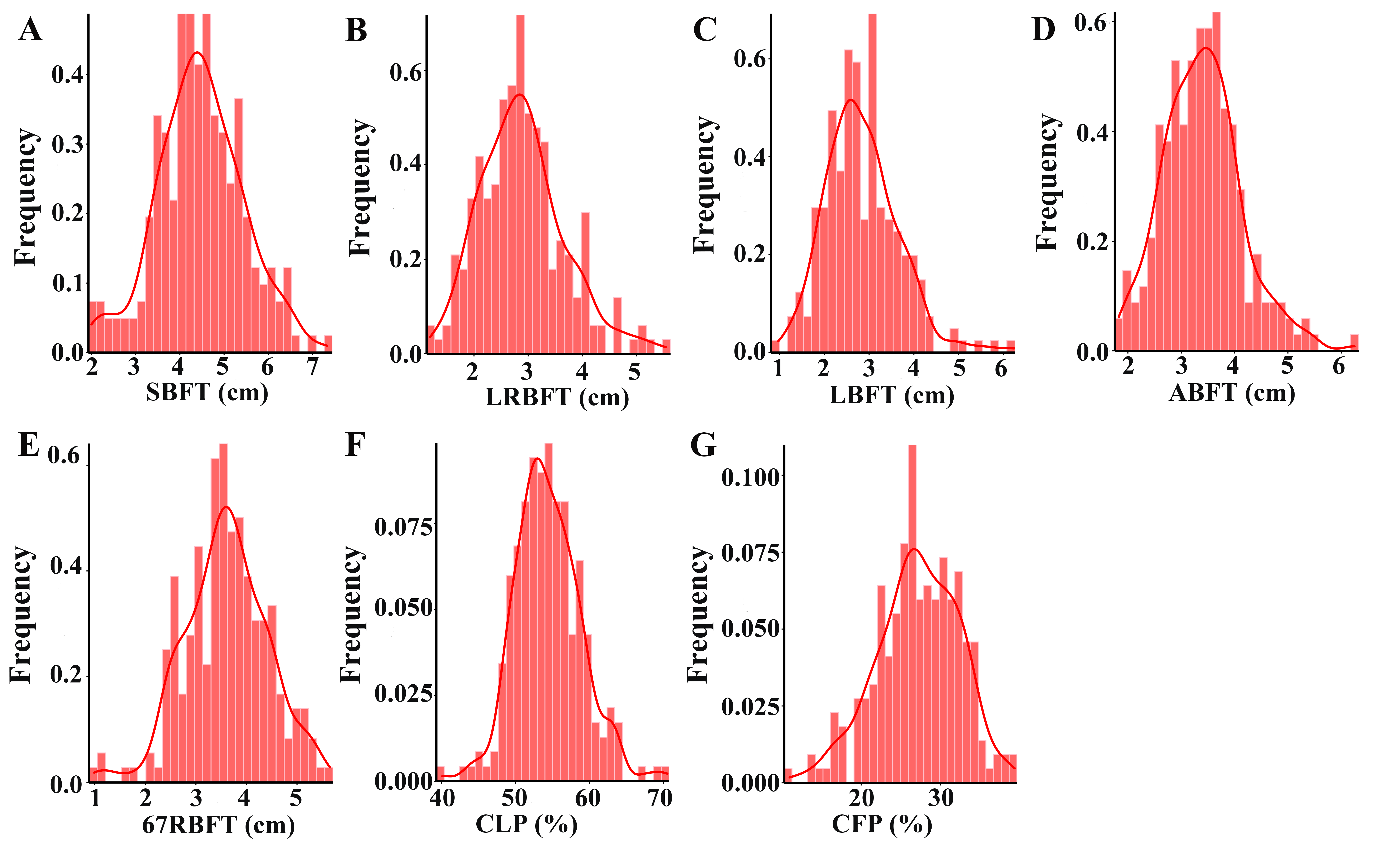

Supplement: Supplementary file 1 — Additional file 1: Figure S1. Frequency distribution histogram for seven fatness-related traits, including five BFTs, CLP and CFP. A SBFT. B LRBFT. C LBFT. D ABFT. E 67RBFT. F CLP. G CFP. [file 12864_2022_8827_MOESM1_ESM.tif]

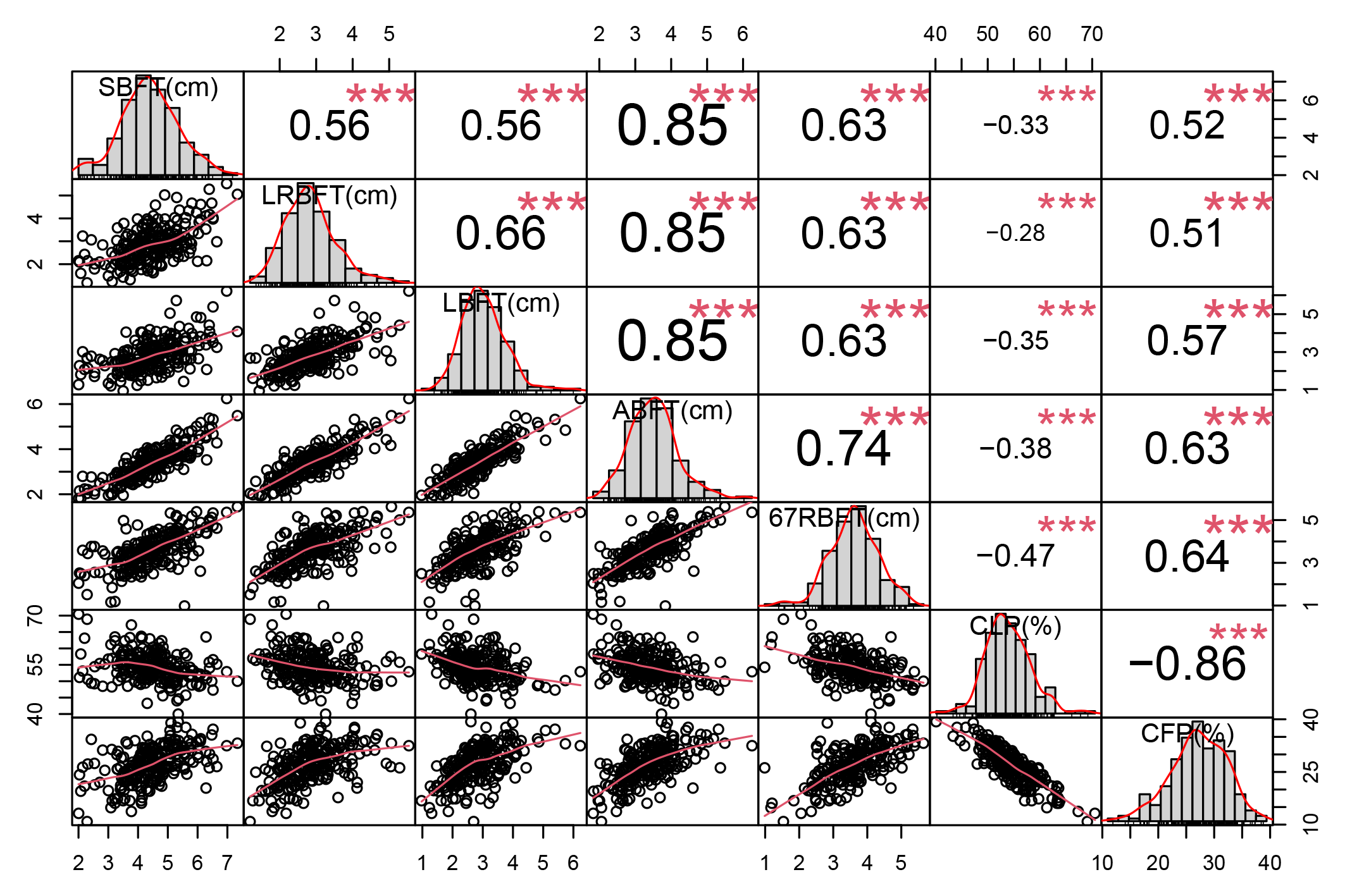

Supplement: Supplementary file 2 — Additional file 2: Figure S2. The phenotypic correlation for seven fatness-related traits, including five BFTs, CLP and CFP. The values in the box represented the phenotypic correlation of the traits. Negative values represented negative correlation, and positive values represented positive correlation. *significant at P<0.05, **significant at P<0.01, ***significant at P<0.001. All of the phenotypic correlation coefficients were significant with P < 0.05. [file 12864_2022_8827_MOESM2_ESM.tif]
