# Supplementary material for: Genome-wide association study identifying genetic variants associated with carcass backfat thickness, lean percentage and fat percentage in a four-way crossbred pig population using SLAF-seq technology
Source: BMC Genomics. 2022 Aug 15;23:594. doi: 10.1186/s12864-022-08827-8 (PMC9380336; doi:10.1186/s12864-022-08827-8)
Supplement: Supplementary file 3 — Additional file 3: Table S1. Distribution of SLAF tags and polymorphism SLAF tags on Sus Scrofa chromosomes. [file 12864_2022_8827_MOESM3_ESM.docx]

**Table S1.** Distribution of SLAF tags and polymorphism SLAF tags on *Sus Scrofa* chromosomes.

| **Chromosome ID** | **SLAF number** | **Polymorphic SLAF^1^** |
| --- | --- | --- |
| 1 | 28103 | 25348 |
| 2 | 15457 | 14803 |
| 3 | 14246 | 13770 |
| 4 | 13613 | 13127 |
| 5 | 10322 | 9920 |
| 6 | 18676 | 17934 |
| 7 | 13245 | 12825 |
| 8 | 13447 | 12891 |
| 9 | 14516 | 11357 |
| 10 | 7255 | 7092 |
| 11 | 7619 | 7403 |
| 12 | 6486 | 6278 |
| 13 | 21264 | 20019 |
| 14 | 15696 | 15048 |
| 15 | 14090 | 13007 |
| 16 | 8142 | 7849 |
| 17 | 7107 | 6875 |
| 18 | 5991 | 5740 |
| X | 9793 | 8674 |
| Y | 650 | 266 |
| Scaffold | 16 | 13 |
| Total | 245734 | 230239 |

^1^Polymorphic SLAF exhibited sequence polymorphisms between different accessions

*SLAF* Specific-locus amplified fragment
