# Supplementary material for: Genome-wide association study identifying genetic variants associated with carcass backfat thickness, lean percentage and fat percentage in a four-way crossbred pig population using SLAF-seq technology
Source: BMC Genomics. 2022 Aug 15;23:594. doi: 10.1186/s12864-022-08827-8 (PMC9380336; doi:10.1186/s12864-022-08827-8)
Supplement: Supplementary file 4 — Additional file 4: Table S2. The information of shared SNPs for fatness-related traits. [file 12864_2022_8827_MOESM4_ESM.docx]

| **Trait^1^** | **SNP^2^** | **Location^3^** | **Gene** |
| --- | --- | --- | --- |
| LRBFT, 67RBFT | rs332294996 | SSC3:34308396 | NA |
| SBFT, ABFT |  | SSC7:21392136 | *POM121L2*, *ZNF184*, *ZNF391* |
| LRBFT, ABFT | rs320451735 | SSC7:21466553 | *POM121L2*, *ZNF184*, *ZNF391* |
| SBFT, ABFT | rs1112937671 | SSC7:29486003 | *COL21A1* |
| SBFT, LRBFT,  LBFT, ABFT |  | SSC7:29503670 | *COL21A1* |
| CLP, CFP | [rs320036825](http://asia.ensembl.org/Sus_scrofa/Variation/Explore?db=core;g=ENSSSCG00000001500;r=10:46524739-46524739;source=dbSNP;v=rs320036825;vdb=variation;vf=2219439) | SSC10:46524739 | *FAM171A1* |
| CLP, CFP | [rs329489266](http://asia.ensembl.org/Sus_scrofa/Variation/Explore?db=core;g=ENSSSCG00000001500;r=11:47266057-47266057;source=dbSNP;v=rs329489266;vdb=variation;vf=11493458) | SSC11:47266057 | NA |
| CLP, CFP | [rs81261044](http://asia.ensembl.org/Sus_scrofa/Variation/Explore?db=core;g=ENSSSCG00000001500;r=11:47266119-47266119;source=dbSNP;v=rs81261044;vdb=variation;vf=236787) | SSC11:47266119 | NA |
| ABFT, 67RBFT |  | AEMK02000449.1:179574 | *OR4P4L*, *OR4C13L*,  *OR5D14*, *OR5D13L* |

**Table S2** The information of shared SNPs for fatness-related traits

^1^*SBFT* Backfat thickness at the shoulder *LRBFT* Backfat thickness at the last rib *LBFT* Backfat thickness at the last lumbar *ABFT* Average backfat thickness *67RBFT* Backfat thickness at 6-7 ribs *CLP* Carcass lean percentage *CFP* Carcass fat percentage ^2^SNP rs ID from Ensembl ^3^Positions of the significant SNP according to the *Sus Scrofa* Build 11.1 assembly *SSC* *Sus Scrofa* chromosome
