# Supplementary material for: Genome-wide association study identifying genetic variants associated with carcass backfat thickness, lean percentage and fat percentage in a four-way crossbred pig population using SLAF-seq technology
Source: BMC Genomics. 2022 Aug 15;23:594. doi: 10.1186/s12864-022-08827-8 (PMC9380336; doi:10.1186/s12864-022-08827-8)
Supplement: Supplementary file 6 — Additional file 6: Table S4. The description and GO annotation of the gene with 100 kb upstream and downstream of the significant SNP. [file 12864_2022_8827_MOESM6_ESM.docx]

**Table S4** The description and GO annotation of the gene with 100 kb upstream and downstream of the significant SNP

| **Genes** | **Descriptions** | **GO annotation^1^** |
| --- | --- | --- |
| *ZNF391* | Zinc finger protein 391 | BP: regulation of transcription, DNA-templated (GO:0006355); MF: metal ion binding (GO:0046872); nucleic acid binding (GO:0003676); |
| *ZNF184* | Zinc finger protein 184 | BP: regulation of transcription, DNA-templated (GO:0006355); MF: DNA binding (GO:0003677); metal ion binding (GO:0046872); |
| *COL21A1* | Collagen type XXI alpha 1 chain | CC: collagen trimer (GO:0005581); extracellular matrix (GO:0031012); |
| *GRM4* | Glutamate metabotropic receptor 4 | BP: activation of MAPK activity (GO:0000187); MF: group III metabotropic glutamate receptor activity (GO:0001642); |
| *NUDT3* | Nudix hydrolase 3 | MF: magnesium ion binding (GO:0000287); diphosphoinositol-polyphosphate diphosphatase activity (GO:0008486); CC: extracellular exosome (GO:0070062); |
| *HMGA1* | High mobility group protein HMG-I | NA |
| *PGM2L1* | Phosphoglucomutase 2 like 1 | BP: carbohydpercentage metabolic process (GO:0005975); phosphorylation (GO:0016310); MF: intramolecular transferase activity, phosphotransferases (GO:0016868); glucose-1,6-bisphosphate synthase activity (GO:0047933); |
| *PLBD2* | Phospholipase B-like 2 | BP: lipid catabolic process (GO:0016042); MF: hydrolase activity (GO:0016787); |
| *FAM171A1* | Family with sequence similarity 171 member A1 | BP: regulation of cell shape (GO:0008360); CC: integral component of membrane (GO:0016021); |
| *KIAA1217* |  | BP: embryonic skeletal system development ([GO:0048706](https://www.ebi.ac.uk/QuickGO/term/GO:0048706)); |

^1^*BP* Biological Process *MF* Molecular Function *CC* Cellular Component
